# Type 1 Diabetes Mellitus-Associated Genetic Variants Contribute to Overlapping Immune Regulatory Networks

**DOI:** 10.3389/fgene.2018.00535

**Published:** 2018-11-21

**Authors:** Denis M. Nyaga, Mark H. Vickers, Craig Jefferies, Jo K. Perry, Justin M. O’Sullivan

**Affiliations:** ^1^The Liggins Institute, The University of Auckland, Auckland, New Zealand; ^2^Starship Children’s Health, Auckland, New Zealand

**Keywords:** Type 1 diabetes, genome-wide association studies (GWAS), genetic variation, genome organization, expression quantitative trait loci (eQTL), autoimmunity

## Abstract

Type 1 diabetes (T1D) is a chronic metabolic disorder characterized by the autoimmune destruction of insulin-producing pancreatic islet beta cells in genetically predisposed individuals. Genome-wide association studies (GWAS) have identified over 60 risk regions across the human genome, marked by single nucleotide polymorphisms (SNPs), which confer genetic predisposition to T1D. There is increasing evidence that disease-associated SNPs can alter gene expression through spatial interactions that involve distal loci, in a tissue- and development-specific manner. Here, we used three-dimensional (3D) genome organization data to identify genes that physically co-localized with DNA regions that contained T1D-associated SNPs in the nucleus. Analysis of these SNP-gene pairs using the Genotype-Tissue Expression database identified a subset of SNPs that significantly affected gene expression. We identified 246 spatially regulated genes including *HLA-DRB1*, *LAT*, *MICA*, *BTN3A2*, *CTLA4*, *CD226*, *NOTCH1*, *TRIM26*, *PTEN*, *TYK2*, *CTSH*, and *FLRT3*, which exhibit tissue-specific effects in multiple tissues. We observed that the T1D-associated variants interconnect through networks that form part of the immune regulatory pathways, including immune-cell activation, cytokine signaling, and programmed cell death protein-1 (PD-1). Our results implicate T1D-associated variants in tissue and cell-type specific regulatory networks that contribute to pancreatic beta cell inflammation and destruction, adaptive immune signaling, and immune-cell proliferation and activation. A number of other regulatory changes we identified are not typically considered to be central to the pathology of T1D. Collectively, our data represent a novel resource for the hypothesis-driven development of diagnostic, prognostic, and therapeutic interventions in T1D.

## Introduction

Type 1 diabetes (T1D) is a chronic immune-mediated disease characterized by the progressive loss of insulin-secreting pancreatic beta cells, and the incidence is slowly rising worldwide (Insel et al., 2015). Well-powered genome-wide association studies (GWAS) have identified more than 60 susceptibility regions to T1D across the human genome, which are marked by single-nucleotide polymorphisms (SNPs) (Ram et al., 2016). The major heritable risk (∼50%) for T1D is conferred by SNPs located within the human leukocyte antigen (HLA) region (Ounissi-Benkalha and Polychronakos, 2008). To date, however, the functional roles of most T1D-associated genetic variants is yet to be determined. Notably, 90 percent of these genetic variants fall outside coding regions (Ward and Kellis, 2012), and therefore, their biological role in the pathogenesis of diseases is not clear. However, there is growing evidence supporting a putative role for these non-coding variants in the regulation of gene expression, as the majority of SNPs fall within regulatory loci such as enhancer regions (Guo et al., 2015; Javierre et al., 2016; Ram and Morahan, 2017).

Classically, GWAS-associated SNPs which fall outside the coding regions of genes have been assumed to affect the most “biologically relevant” or closest genes (McGovern et al., 2016). A fundamental problem with this assumption is that many intergenic SNPs may influence the expression of genes which are quite distal (Farh et al., 2014; Schoenfelder et al., 2015). Indeed, our enhanced understanding of chromosome architecture and nuclear organization over recent years has shown that interactions with regulatory regions (e.g., insulators and enhancers) regularly bypass the closest genes and are associated with changes in gene transcript levels of genes located large genomic distances away. These interactions may occur within the same, or on different chromosomes (Bulger and Groudine, 2011; Sanyal et al., 2012; Javierre et al., 2016).

Most studies on spatial genomics ignore the impact of these distal-regulatory chromosome interactions despite increasing evidence that genetic polymorphisms as identified by GWAS can alter the expression of genes through distal spatial interactions in a tissue- and development-specific manner (Fehrmann et al., 2011; Schierding et al., 2015; Fadason et al., 2017). For example, Fadason et al. (2017) identified spatially regulated genes (e.g., *IRS1*, *ADIPOQ*, *FADS2*, *PPA2*, and *WFS1*) within tissues and pathways that are recognized as important for type 2 diabetes progression. This was achieved by integrating information on spatial chromatin organization (Rao et al., 2014), and functional data [i.e., expression quantitative trait loci (eQTL)] to assign SNPs to the genes they control. This finding highlights the importance of integrating an understanding of the spatial genomic context into analyses to discover the fundamental mechanisms underlying gene regulation (Ottaviani et al., 2012; Javierre et al., 2016; Willmann et al., 2016; Won et al., 2016).

We hypothesized that T1D-associated SNPs, and particularly the SNPs along the immune-associated HLA locus, contribute to disease pathogenesis by deregulating expression of genes in a tissue-specific manner. In the present study, we used the Contextualize Developmental SNPs in 3D (CoDeS3D) algorithm to perform a combined spatial and functional eQTL analysis to assign T1D-associated genetic variants to the genes they regulate. We identified interconnected regulatory networks of spatially associated T1D eQTLs that affect immune pathways (adaptive immune signaling and immune-cell proliferation and activation). We demonstrate that T1D-associated SNPs have effects in tissues that are not classically associated with T1D, such as liver, brain hypothalamus, and adrenal. These findings provide a novel platform for the development of novel diagnostic and therapeutic interventions.

## Materials and Methods

### Identification of T1D Associated SNPs

Single nucleotide polymorphisms associated with T1D (*p* ≤ 9.0 × 10^-6^) were selected from the manually curated Catalog of Published Genome-Wide Association Studies (MacArthur et al., 2017)^1^ (November 3, 2017) (Supplementary File 1). These SNPs (11 HLA and 169 non-HLA risk SNPs) represent the ∼60 susceptibility regions that are typically associated with T1D. Equally sized sets of control SNPs were randomly selected from the SNP database (dbSNP database, Build 151; November 10, 2017) using a Python script.

### Regulatory SNP–Gene Interactions and eQTLs Analyses

We identified genes whose transcript levels depend on the identity of the T1D-associated SNP using the CoDeS3D algorithm (GitHub^2^) (Fadason et al., 2017). Initially, the modular python scripts that comprise CoDeS3D use 1 kb resolution Hi-C contacts from non-synchronized immortalized human cell lines [i.e., IMR90 (CCL-186), HMEC (CC-2551), NHEK (192627), KBM7, HUVEC] (Rao et al., 2014) to identify spatial co-localization of two DNA regions, one of which is marked by a SNP. These spatially associating genomic regions are not limited to adjacent regions within the linear DNA sequence (Fadason et al., 2017).

Next, data from the Genotype-Tissue Expression (GTEx) database (version 7^3^; retrieved on November 19, 2017) (Fadason et al., 2017) is incorporated to address whether spatially associated T1D-SNPs are associated with changes in the transcript levels (eQTLs) of the spatially associated genes. The gene list and DNA locations are based on the hg19/GRCh37 human genome reference. The CoDeS3D analysis identifies: (a) SNP-gene pairs that spatially co-localize within the nucleus; (b) SNP-gene pairs that are expression QTLs; and (c) the tissues in which the eQTL is significant, using the Benjamini–Hochberg correction for multiple testing (FDR, *q* < 0.05) (Benjamini and Hochberg, 1995). Although the multiple testing burden of eQTL mapping can bias or misinterpret results, our FDR threshold (*q* < 0.05) has been demonstrated to identify biologically significant associations consistently (e.g., Schierding et al., 2015; Fadason et al., 2017). *Cis*-expression QTL SNPs were defined as occurring within loci <1 Mb. By contrast, *trans*-expression QTL SNPs were defined as occurring between loci >1 Mb apart, or on different chromosomes.

### Gene Ontology (GO), Pathway Analysis, and Functional Prediction

We used web-based applications of Gene Ontology (GO^4^; accessed April 3, 2018) and the Reactome Pathway Database (version 64^5^; accessed April 3, 2018) to annotate significant eGenes (genes regulated by loci marked by the eQTL SNPs) for biological and functional enrichment (Ashburner et al., 2000; Croft et al., 2014; Carbon et al., 2017; Fabregat et al., 2018). The enrichment analysis were performed by standard methods (detailed in Ashburner et al., 2000; Fabregat et al., 2018) using a background set of human proteins to reveal significant enrichments adjusted for multiple comparisons at an FDR threshold *q* < 0.05.

Testing for potential functional regulatory SNPs within the list of T1D-associated SNPs was performed using the sequence-based deep learning-based sequence analyzer-DeepSEA (Zhou and Troyanskaya, 2015). The algorithm integrates a training set of genome-wide chromatin profiles based on the Roadmap Epigenomics and ENCODE datasets (Dunham et al., 2012; Kundaje et al., 2015). Potential regulatory sites are predicted based on over-lap with histone-mark profiles, transcription factor binding, and DNase I sensitivity sites.

All statistical analyses were performed using R software (version v3.4.2) (R Core Team, 2014).

## Results

### T1D-Associated Variants Form a Gene Regulatory Network

**FIGURE 1:**
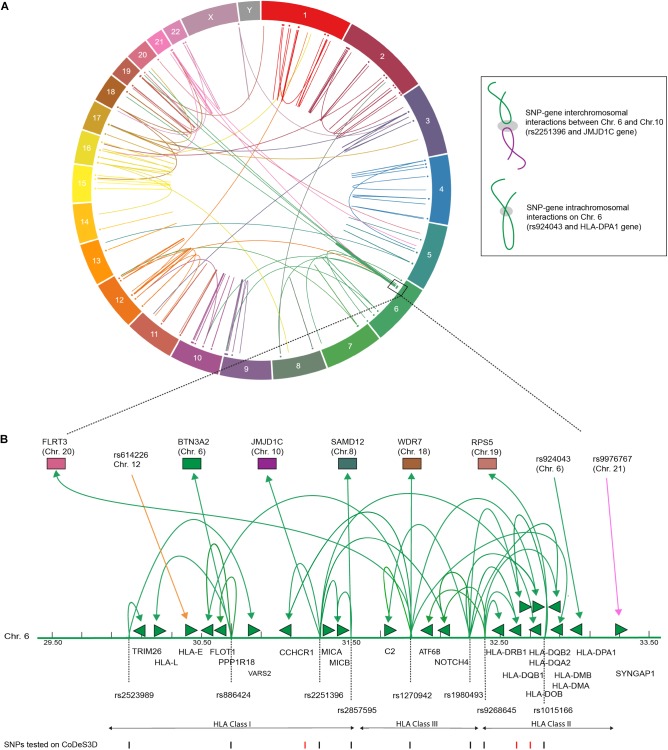
T1D associated SNPs form an integrated gene regulatory network. **(A)** Circos plot showing eQTL associations between T1D SNPs and eGenes that overlap with Hi-C data. Data tracks: chromosome labels (outer-most ring); and a scatter plot of relative SNP positions. Link lines represent significant SNP-gene interactions at FDR *q* < 0.05 (Supplementary Table 2). The inset illustrates a *cis*- and *trans*-eQTL. The gray ellipsoid represents the unknown factors that are responsible for mediating the physical interaction. **(B)** T1D associated genetic variants are involved in *cis*- and *trans*-eQTLs that enter and emerge from the HLA locus. Significant *cis*- and *trans*-eQTLs are annotated by lines with arrows, green arrows heads denoted direction of the regulatory effect. Genes within the HLA locus are annotated as arrows, which indicate transcriptional direction. SNP positions are marked as lines (black – lines indicate significant SNPs, FDR < 0.05). eGenes affected by *trans*-eQTLs are colored according to chromosome number as in **(A)**.

We identified 232 *cis*- and 66 *trans*-eQTLs, at an FDR of *q* < 0.05 for SNPs associated with T1D (Supplementary Table 1). The functional physical interactions between T1D-associated SNPs and eGenes (i.e., the genes whose transcript levels are associated with the identity of the nucleotide at the SNP position) were represented in a circos plot (Figure 1). We observed a series of *trans*-eQTLs that connect into and out of the HLA locus (Figure 1B). The observed *cis*- and *trans*-eQTL network for T1D associated SNPs is consistent with a functional role for SNPs in modulating gene expression profiles, rather than protein sequences, that predispose an individual to the development of T1D (Størling and Pociot, 2017). The identification of 66 *trans*-eQTLs, which by definition interact with regions >1 Mb apart or on different chromosomes, reinforces the importance of not identifying eGenes on the basis of linear proximity in the absence of observable transcriptional effects due to the cell and tissue-specific contexts underlying gene expression.

A Monte Carlo experiment was performed to test for eQTL enrichment using randomly selected SNPs from the SNP database (dbSNP build 151; November 10, 2017). The Monte Carlo permutation demonstrate that T1D-associated SNPs have significantly (*t*-test *p*-value <0.0001) more SNP-gene connections than equally sized sets of randomly selected dbSNPs (Supplementary Table 1). These results are consistent with previous comparisons of randomly selected SNPs and those specific for type-2 diabetes or obesity using CoDeS3D (Fadason et al., 2017).

We identified 25 SNP-eQTLs (associated with transcript levels of 46 *cis*- and 25 *trans*-eGenes) that were predicted (DeepSEA score <0.05) to have functional regulatory roles within the genome (Supplementary Table 3). A comparison of the ratios of *cis*:*trans* interactions between the 25 DeepSEA predicted regulatory SNP-eQTLs and the 88 non-regulatory SNP-eQTLs, identified an enrichment (*p*-value of 0.00576; Fisher exact test) for trans interactions involving the regulatory SNP-eQTLs. Collectively, these results are consistent with evidence that inter-genic SNPs associated with disease phenotypes mark gene regulatory regions (Ernst et al., 2011).

We used Gene Ontology (GO; see text footnote 4) and the Reactome Pathway Database to annotate T1D-eGenes (both *cis*- and *trans*-eGenes) for biological and functional enrichment. The T1D-eGenes were significantly enriched (FDR, *q* < 0.05) for biological processes and canonical pathways associated with antigen processing and presentation; immune-cell activation (T lymphocytes activity); programmed death signaling; and cytokine signaling (Table 1). Our observations are consistent with the T1D-associated variants modifying the expression of genes that are interconnected (directly or indirectly) through networks that form part of immune system pathways (adaptive immune signaling, and immune-cell proliferation and activation). This agrees with previous observations (Newman et al., 2017; Ram and Morahan, 2017), but does not unequivocally prove that variations in these pathways directly contribute to the etiology of T1D.

**Table 1:** Gene ontologies and pathways enrichment for T1D-eGenes.

^∗^Gene Ontologies (biological process enrichment)	Genes	FDR
Antigen processing and presentation (GO:0019882)	18	8.86E-07
Regulation of immune response (GO:0050776)	35	2.14E-05
Antigen processing and presentation of exogenous antigen (GO:0019884)	14	5.55E-05
Regulation of T cell activation (GO:0050863)	18	6.38E-05
Positive regulation of leukocyte cell-cell adhesion (GO:1903039)	15	6.51E-05
Cell surface receptor signaling pathway (GO:0007166)	52	2.31E-04
Regulation of lymphocyte activation (GO:0051249)	19	1.05E-03
Response to stimulus (GO:0050896)	122	2.19E-03
Interferon-gamma-mediated signaling pathway (GO:0060333)	7	8.37E-03
Regulation of mononuclear cell proliferation (GO:0032944)	10	2.63E-02

**^∗∗^Reactome pathways enrichment**	**Entities found¶**	**FDR**

Translocation of ZAP-70 to immunological synapse (R-HSA-202430)	18	2.43E-14
Phosphorylation of CD3 and TCR zeta chains (R-HSA-202427)	18	2.43E-14
Generation of second messenger molecules (R-HSA-202433)	19	2.43E-14
PD-1 signaling (R-HSA-389948)	18	2.43E-14
Co-stimulation by the CD28 family (R-HSA-388841)	20	1.04E-11
MHC class II antigen presentation (R-HSA-2132295)	23	3.16E-11
Downstream TCR signaling (R-HSA-202424)	21	7.08E-11
TCR signaling (R-HSA-202403)	22	1.65E-10
Interferon gamma signaling (R-HSA-877300)	27	8.26E-10
Interferon signaling (R-HSA-913531)	29	5.58E-07

*^∗^Significant gene ontologies (biological process) and ^∗∗^Reactome pathways for T1D-eGenes were derived from http://www.geneontology.org/ and Reactome Pathway Database (version 64, https://reactome.org/), respectively, (FDR ≤ 0.05) (Ashburner et al., 2000; Croft et al., 2014; Carbon et al., 2017; Fabregat et al., 2018). ^¶^The number of mapped identifiers corresponding to the pathway for the selected molecular type. PD-1, programmed cell death protein-1; TCR, T-cell receptor; ZAP-70, Zeta-chain-associated protein kinase 70; MHC, major histocompatibility complex. A complete summary of gene ontologies and pathway enrichments is presented in Supplementary File 1.*

### T1D-Associated SNPs Across the HLA Locus Affect Transcript Levels of Genes in *cis* and *trans*

**FIGURE 2:**
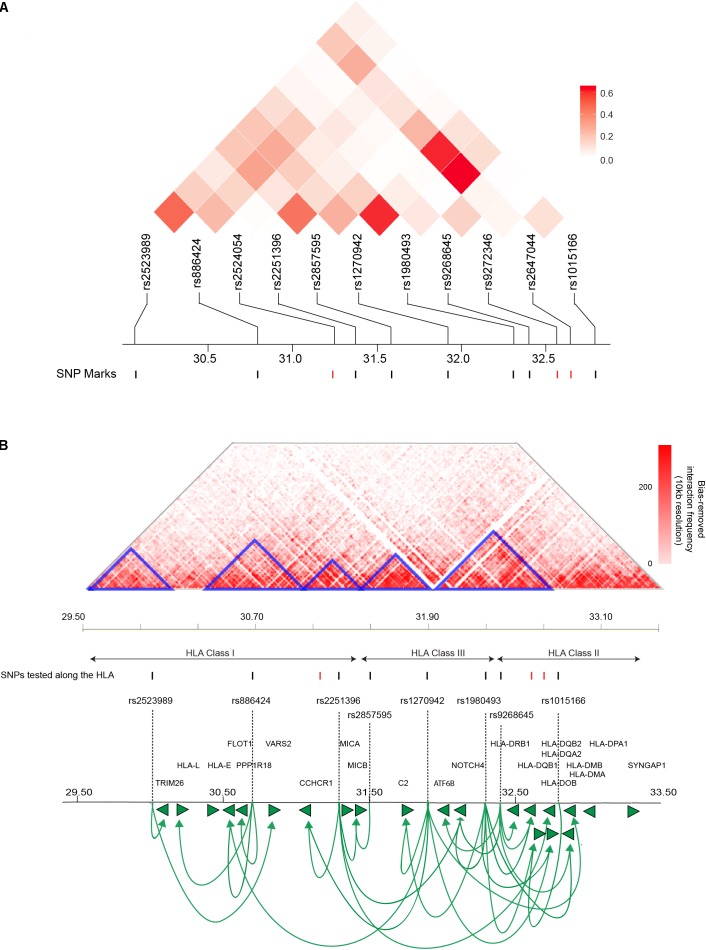
T1D-associated variants along the HLA locus affect gene expression within and outside of the locus. **(A)** Linkage disequilibrium (LD) plots of T1D-associated SNPs along the HLA locus amongst people with Western European ancestry (CEU). The squares within the heat map represent the LD (*R*^2^) value between every two variants. The weak LD (*R*^2^ ≤ 0.6) indicates that SNPs are infrequently co-inherited and contribute to disease development independently. SNPs with significant eQTLs are represented by black marks (FDR ≤ 0.05). **(B)** An interaction frequency heat map of intra-chromosomal contacts across the HLA locus (∼4 Mb) captured in the human lymphoblastoid cell line GM12878 at 10 kb resolution (Rao et al., 2014). The heat map color represent the levels of normalized interaction frequencies and triangles illustrate topological association domains (TADs). SNPs that show significant and non-significant eQTLs are denoted by black and red marks, respectively (FDR ≤ 0.05). Loops (lines with arrows) represent interactions between SNPs and genes that are associated with differential expression. Green arrows heads denoted direction of the regulatory effect. The illustrated gene map is as in Figure 1 **(B)**. [The heat map matrix of pairwise LD was plotted at https://ldlink.nci.nih.gov/. Hi-C interaction frequency heat maps were plotted at http://kobic.kr/3div/ (Yang et al., 2018)].)

There is a possibility that the T1D-associated SNPs located across the HLA locus are associated with the transcript levels of multiple genes or that they combine to regulate the expression of a single strong risk allele. To distinguish between these two possibilities, we analyzed the linkage disequilibrium (LD) profile for the HLA based genetic variants (11 SNPs) associated with T1D amongst people with Western European ancestry (CEU). We observed maximal linkage value of *R*^2^ ≤ 0.6 (Figure 2A). An *R*^2^ > 0.8 is generally accepted as indicating robust linkage (Smith, 2005). Therefore, the linkage we observed between the SNPs we tested was relatively weak (*R*^2^ ≤ 0.6). The inter-eQTL LD we observed is consistent with the majority of the T1D-associated SNPs contributing to the development of disease independently. However, even within the low levels of LD we observed, it is notable that there are two predominant examples of long-distance LD between rs1980493-rs1270942, and rs1270942-rs2647044/rs1980493-rs2647044 (Figure 2A). Long-distance LD has been characterized across the human genome and previously hypothesized to be associated with gene regulation (Koch et al., 2013).

The role of genome structure in gene regulation is widely considered to be represented in the local chromosome structure observed within topologically associating domains (TADs), chromosomal regions which physically interact frequently more than their genomic neighbors (Schmitt et al., 2016). Therefore, we determined how the HLA eQTLs were positioned with respect to TADs and local chromatin structure within the human lymphoblastoid cell line GM12878, at 10 kb resolution (Figure 2B). The GM12878 lymphoblastoid cell line was used to examine the 3D genome architecture since it has the densest contact map, containing approximately 4.9 billion Hi-C captured contacts (Rao et al., 2014). Significant eQTLs involving rs2523989, rs886424, rs2251396, rs2857595, rs1980493, rs1015166, rs1270942, and rs9268645 occurred within TADs that were located across the HLA class I, II, and III regions (Figure 2B).

Genetic variant rs1270942 was located at, or in close proximity to, the TAD boundary (Figure 2B) and was observed to spatially regulate the expression of genes in both the HLA class I and II regions. Interestingly, rs1270942 was predicted to be regulatory (DeepSEA) (Supplementary Table 3). SNP rs9268645 was not predicted to be a regulatory SNP by DeepSEA (Supplementary Table 3). Yet, rs9268645 falls in a boundary region and is an eQTL for HLA-DRB1, whose expression was reported to involve regulation by CCCTC-binding factor (CTCF) through long-distance chromatin looping (Majumder et al., 2008). The observed regulatory activity of rs9268645 is consistent with observations that TAD boundary sites contain elevated levels of the transcription factor CTCF.

Three SNPs within the HLA region (rs2524054, rs9272346, and rs2647044) were not found to be involved in eQTLs in our analysis (Figure 2B). None of these SNPs (rs2524054, rs9272346, and rs2647044) were predicted to have regulatory functional roles using DeepSEA algorithm (Supplementary File 1). Notably, rs9272346 and rs2524054 fall within coding regions, while rs2647044 is intergenic. This indicates that there are other mechanisms through which these genetic variants contribute to T1D disease development. Collectively, our results are consistent with: (a) T1D-associated variants within proximal and distal regulatory regions directly affecting expression (i.e., transcript levels) within and outside of the HLA locus; and (b) interactions between functional polymorphisms and gene regulatory elements being associated with inheritance (i.e., long-distance LD).

### Expression QTLs Contribute to Tissue-Specific Effects in Autoimmune T1D

**FIGURE 3:**
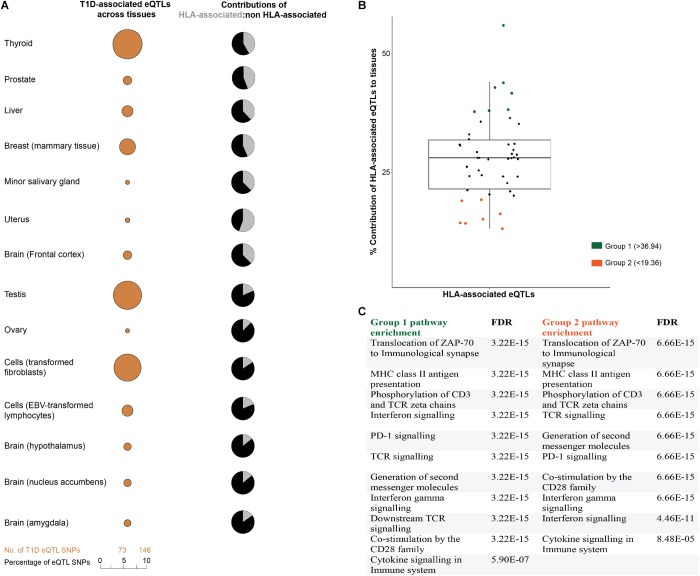
T1D-associated eQTL effects are tissue-specific. **(A)** T1D-associated eQTLs are differentially distributed across human tissues. The differential distribution is epitomized by the relative proportions of HLA and non-HLA associated eQTLs in different tissues. A complete summary of all GTEx tissues with significant eQTLs (FDR ≤ 0.05) is presented in Supplementary Figure 1. **(B)** The relative contributions of HLA associated T1D eQTLs to tissue specific effects. Relative contribution was calculated (HLA: total eQTLs for a tissue expressed as a percentage). The mean HLA contribution was 28.16 ± 8.79%. **(C)** eGenes within tissues with high or low HLA contributions (i.e., ±1 SD from the mean) were enriched for biological pathways associated with immune pathways. Biological pathway enrichment was performed using the Reactome pathways database (Fabregat et al., 2018), with significant (FDR ≤ 0.05) for immune response pathways.

We hypothesized that disease-relevant biological processes are fundamentally dependent on mRNA levels, with the cross-tissue variability of gene expression providing an important avenue for understanding disease etiologies. Therefore, we analyzed the tissue-specific contributions of T1D-associated eQTLs to tissues. Consistent with our hypothesis, eQTL effects were distributed differently across human tissues (Figure 3A). Tibial artery and lower leg skin tissues have been linked to peripheral arterial disease in diabetic patients (American Diabetes Association, 2003; Thiruvoipati, 2015). Both tibial artery and lower leg skin were observed to have the highest proportion; while brain substantia nigra tissue displayed the lowest proportion of eQTLs (Supplementary Figure 1).

We compared the proportions of HLA and non-HLA associated eQTLs across different tissues and identified two tissue groups (group 1 and 2) that were located one SD from the mean (HLA: total eQTL percentage of 28.16 ± 8.79) (Figure 3B). Analysis of the eGenes within these groups, using Reactome pathways database, identified biological enrichment within antigen presentation, programmed cell death signaling, T-cell receptor signaling, and interferon gamma signaling) for both groups of tissues (FDR ≤ 0.05; Figure 3C). The identification of changes in transcript levels involved in immune activation, signaling, and response pathways in tissues that are not traditionally associated with T1D pathology is notable. This may indicate that dysregulation of the immune-associated signals within these tissues contributes to the onset or development of T1D. Collectively, we observed cross-tissue concordance for T1D-associated eQTL effects (either HLA or non-HLA associated; Figure 3B), which is consistent with multiple tissues and specific biological pathways impacting on T1D progression (Mei et al., 2017).

## Discussion

The etiology of T1D is hypothesized to involve T cell-mediated destruction of the insulin-producing pancreatic islet beta cells, leading to complete insulin loss (Insel et al., 2015). We have integrated genetic variation, 3D genome organization and functional analyses (eQTLs) to better understand the downstream effects of SNPs associated with T1D. Our findings identify overlapping regulatory networks that contribute to adaptive immune signaling, immune-cell proliferation and activation in a tissue-specific manner. We contend that the integration of mixed “omics” datasets into the functional interpretation of T1D-associated SNPs has identified novel pathways and tissue-specific functional genetic loads that represent high priority targets for clinical investigation into the developmental windows and effects that contribute to T1D.

Our analyses identify associative functional effects of T1D GWAS-associated SNPs in regulating the tissue-specific expression of target genes either through *cis*- or *trans*-interactions. Importantly, Hi-C interaction patterns captured from cells demonstrate permissible contacts that can occur at a detectable frequency across populations of cells in human tissues (Nagano et al., 2013). Moreover, it is widely recognized that topologically associated domains captured in cell lines and lineages are highly conserved (Dixon et al., 2015). We therefore assert that the high-resolution Hi-C data from the immortalized human cell lines [i.e., derived from Rao et al. (2014)] represents interactions that can form within the human genome in different tissues. However, a potential limitation of our study is that it is not possible to identify all potential chromatin interactions across different cell types, thus there is a possibility that several tissue-specific chromatin interactions may be missing. As such, future work should integrate tissue and developmental stage specific chromatin architecture into analyses to identify all possible interactions.

Contextualize Developmental SNPs in 3D does not take into account the linkage disequilibrium of genetic variants, rather the resolution is based on the interactions that the restriction fragments were captured in the Hi-C experiments that determine genome organization. Analysis by CoDeS3D does not designate the tested SNPs as being causal, as other SNPs located within the same restriction fragment that are in strong linkage are not separable using the algorithm (Fadason et al., 2017). However, if strongly linked SNPs are located on different restriction fragments then their effects are separable as the characterization of the eQTLs is dependent upon the genes that the restriction enzymes were captured interacting with in the nucleus.

Not all of the eQTL SNPs that we identified were identified as being regulatory by the DeepSEA machine learning algorithm. We contend that the 25 “functional” SNPs predicted by DeepSEA overlap putative gene regulatory regions (i.e., enhancer and transcription binding sites), while the 88 “non-functional” SNPs are in LD with a regulatory locus within a genomic restriction fragment. Additionally, the 17 SNPs that DeepSEA predicted to be “functional” from within the set of 70 non-significant SNP-eQTLs indicates that: (a) not all tissue and cell-specific chromatin states are represented in the CoDeS3D analysis; and/or (b) the eQTLs within GTEx do not represent all of the developmental stages that are relevant to T1D.

Allelic variations in the antigen presentation mediated by the HLA locus under-pin autoimmune disease (Raj et al., 2016). The low levels of LD observed for the eQTLs within the HLA region indicates that these T1D-associated SNPs contribute to the development of disease independently as they are rarely co-inherited. This is consistent with Barcellos et al. (2009) who identified the existence of multiple, independent disease susceptibility regions within the HLA locus (Barcellos et al., 2009). However, there are notable examples of multiple susceptibility loci within HLA haplotypes combining to influence T1D risk through co-regulation (Figure 2B). It remains to be determined if the T1D-associated genetic variants within the HLA locus disrupt the coordinated chromatin configuration (Raj et al., 2016). However, long-distance regulation involving regulatory loci at TAD boundaries in the HLA locus has been observed previously (Majumder et al., 2008; Majumder and Boss, 2010). Future work should use CRISPR-Cas or Degron based strategies (Sander and Joung, 2014; Guharoy et al., 2016) to empirically confirm the mechanisms by which genetic variations at these loci results in transcriptional changes in order to enable the development of targeted therapeutic or prognostic approaches.

Studies have identified the HLA class II region as a recombination hotspot, resulting in a disrupted LD pattern for a region that is known to exhibit robust linkage (Crawford et al., 2004; Miretti et al., 2005). Population history (i.e., genetic drift), chromosomal recombination activity, and selective pressure could potentially explain the observed long-range LD we observed across the class III – class II HLA regions (Miretti et al., 2005). We contend that understanding the co-inheritance of the eQTL SNP-egene pairs will provide fundamental insights into the associated protection and susceptibility for particular HLA haplotypes in T1D development across different populations (Cucca et al., 2001). This will be particular relevant in populations that are undergoing relatively rapid and high levels of genetic admixture.

Type 1 diabetes-associated eQTL mapping studies have focused on cell-specific effects in immune cell types (e.g., Kasela et al., 2017; Ram and Morahan, 2017). This approach is based on the assumption that these tissues are critical to understanding the development and pathology of T1D. Onengut-Gumuscu et al. (2015) demonstrated that genetic variants associated with T1D are enriched within enhancer sequences of active CD34^+^ stem cells, T and B lymphocytes, and the thymus gland. Notably, the integration of Hi-C data (enhancer-promoter interactions) with T1D-associated SNPs across GTEx tissues in our study implicates dysregulated immune signaling and responses in tissues that are not traditionally considered central to the molecular etiology of T1D. Thus, it remains possible that the functional load that is associated with HLA-associated eQTLs as observed in the uterine tissues reflects the unique immune status of this tissue, and contributes to the development of gestational diabetes mellitus in individuals (Binder et al., 2015). Similarly, adipose tissue has recently been hypothesized to play a role in antigen presentation and modulation of T cells (Huh et al., 2014). Therefore, identifying the developmental windows and how the functional genetic loads within these non-traditional tissues contribute to dysregulated immune activity in T1D will be important in uncovering the trigger(s) for and developmental program of T1D.

The enrichment of T1D-associated *cis*- and *trans*-eGenes within immune response pathways that include: antigen presentation; T lymphocytes and B cell activity; receptor signaling complex; and scaffold activity may explain a component of their contribution to the pathogenesis of T1D. For example, the *BTN3A2* gene product plays an important role in T-cell responses in the adaptive immune response by inhibiting the release of interferon gamma (IFN-γ) from activated T-cells (Messal et al., 2011). Aberrant expression of IFN-γ is associated with a pathogenic role in T1D (Bazzaz et al., 2014). Therefore, it was significant that we observed that *BTN3A2* transcript levels were linked to rs886424, by a spatial *trans*-eQTL, in 32 tissues. The associative role of eQTL-SNPs in the *trans*- (i.e., *NUP93*[rs12708716], *HLA-E*[rs614226], *HLA-DPA1*[rs924043], and *HLA-DQB2*[rs2251396]) and *cis*-regulation (i.e., *TRIM26*[rs2523989] and *TYK2*[rs2304256]) of genes within the interferon signaling pathways raises the possibility that changes in the distal regulation of genes within pathways contributes to disease pathogenesis. The observed associative *trans*-regulation of the *ARHGAP42* gene (a member of the Rho GTPase activating proteins) by rs3184504 in EBV-transformed lymphocytes demonstrates that *trans*-interactions can contribute to a cell-type specific regulatory mechanism. Interestingly, Fairfax et al. (2012) demonstrated a monocyte-specific *trans*-association of *ARHGAP24* gene with the DRB1^∗^04, ^∗^07, and ^∗^09 alleles. Notably, these DRB1 alleles are associated with expression of *HLA-DRB4*, which encodes the DR53 super-antigen in autoimmune disease progression (Heldt et al., 2003).

The *SORBS1* gene (a *trans*-eGene for rs1326934) was observed to be differentially up-regulated in the oesophageal mucosa tissue. The *SORBS1* gene product, sorbin, is involved in insulin function (i.e., signaling and stimulation) and has been implicated in insulin resistance and the pathogenesis of diabetic nephropathy (Lin et al., 2001; Germain et al., 2015). The association between rs3825932 and the upregulation of *CTSH* (a lysosomal cathepsin protease) transcript levels in the pancreas and liver tissues complements a known regulatory role for the protease in beta cell function (Floyel et al., 2014). Specifically, the overexpression of *CTSH* in an insulin secreting beta cell derived cell line (INS-1) has been shown to be anti-apoptotic through a reduction in p38 and JNK activity; and downregulation of the pro-apoptotic factors *Bim*, *DP5*, and *c-Myc* (Floyel et al., 2014). *CTSH* is also involved in the positive regulation of insulin transcription, and is a key regulator of beta cell function during the progression of disease in children with recent-onset T1D (Floyel et al., 2014).

We observed a *trans*-association between rs602662 and the downregulation of *CAMTA1* only within the pancreas. The *CAMTA1* gene product has been demonstrated to play an integral role in the regulation of microRNA profiles (i.e., miR-212/miR-132) and beta cell function, with the differential expression of transcript levels implicated in the pathogenesis of diabetes (Mollet et al., 2016). Collectively our results are consistent with *trans*- and *cis*-regulatory elements, located at or surrounding T1D eQTL-SNPs, acting to control a previously unrecognized network of genes that: (a) modulate the immune response; and (b) affect pancreatic beta cell function and survival. We contend that these spatial-regulatory networks are fundamental to understanding the mechanisms and therapeutic approaches to T1D.

We hypothesize that immune-regulatory mechanisms operate within tolerance ranges, and if not properly regulated they can promote autoimmune reactions. It is therefore intriguing that T1D-associated genetic variants spatially contribute to distinct overlapping regulatory networks that have the potential to modify the development of the autoimmune phenotype. Notably, the direct and indirect interconnectivity of the T1D-associated eQTLs means that they are capable of influencing immune-response genes expression in a tissue and cell-type specific manner. Untangling these effects requires empirical studies that incorporate expression QTL analyses within precision approaches that illuminate the genetic basis of individual immunological responses. These studies should also refine the mapping strategy for the identification of the regulatory connections by extending the Hi-C data to include additional tissue and developmental stage relevant maps of genomic organization. Integration of these data into clinical studies of T1D will enable individualized mechanistic understanding of treatment response, prognosis and disease development.

## Author Contributions

DN ran analyses, interpreted the data, and wrote the first draft. MV, CJ, and JP co-supervised DN, participated in discussions and commented on the manuscript. JO’S directed the study, contributed to data interpretation, and co-wrote the manuscript.

## Conflict of Interest Statement

The authors declare that the research was conducted in the absence of any commercial or financial relationships that could be construed as a potential conflict of interest.

## Footnotes

1 www.ebi.ac.uk/gwas/

2 https://github.com/alcamerone/codes3d

3 https://www.gtexportal.org/

4 http://www.geneontology.org/

5 https://reactome.org/
