# Supplementary material for: Type 1 Diabetes Mellitus-Associated Genetic Variants Contribute to Overlapping Immune Regulatory Networks
Source: Front Genet. 2018 Nov 21;9:535. doi: 10.3389/fgene.2018.00535 (PMC6258722; doi:10.3389/fgene.2018.00535)
Supplement: Supplementary file 1 [file Data_Sheet_1.docx]

**Supplementary Material**

**Type 1 diabetes mellitus-associated genetic variants contribute to overlapping immune regulatory networks**

**Denis M. Nyaga^1^, Mark H. Vickers^1^, Craig Jefferies^1, 2^, Jo K. Perry ^1^,**

**Justin M. O’Sullivan^1,*^**

^1^The Liggins Institute, The University of Auckland, Auckland, New Zealand

^2^Starship Children’s Health, Auckland, New Zealand

***Correspondence:** Justin M. O’Sullivan ([justin.osullivan@auckland.ac.nz](mailto:justin.osullivan@auckland.ac.nz)). The Liggins Institute, University of Auckland, Auckland, New Zealand; Phone: +64 9 9239868

**Supplementary Table 1. Summary of the gene regulatory networks for T1D-associated SNPs analysed by CoDeS3D**

|  | Type 1 diabetes SNPs*  (p ≤ 9.0 x 10^-6^) | Randomly generated SNPs (controls; 80 sets of 180 SNPs)** | | |
| --- | --- | --- | --- | --- |
| N^o^. SNPs | 180 | *Range* | *Mean* | *SEM* |
| N^o^. spatial SNP-gene pairs^§^ | 1467 | 0-117 | 19.48 | 2.89 |
| N^o^. eQTL SNP-gene interactions^¶^ | 1467 | 0-117 | 19.48 | 2.89 |
| N^o^. unique eQTL SNP-gene pairs ^¶¶^ | 298 | 0-22 | 4.7 | 0.46 |
| N^o^. unique cis-eQTL SNP-gene pairs^†^ | 232 | 0-14 | 3.69 | 0.18 |
| N^o^. unique trans-eQTL SNP-gene pairs^††^ | 66 | 0-8 | 1.04 | 0.33 |
| N^o^. eGenes^#^ | 246 | 0-14 | 2.36 | 0.22 |

*T1D-associated SNPs (11 HLA and 169 non-HLA risk SNPs) were identified in the GWAS catalog (download date November 3, 2017; version, v1.0) (Supplementary File 1). § Spatial SNP-gene pairs were total number of spatial connections between SNPs and gene regulatory regions, FDR ≤ 0.05. ¶ the total number of eQTL SNP-gene (pairs) interactions, FDR ≤ 0.05 in at least one GTEx tissue. ¶¶ Non-redundant significant eQTL SNP-genes pairs (FDR ≤0.05). † Cis-eQTL SNPs were defined as occurring in loci <1Mb, FDR ≤0.05. †† trans-eQTL SNPs were defined as occurring between loci > 1Mb apart, or on different chromosomes, with a FDR ≤0.05. # eGenes were those expression observed to be affected by an eQTL SNP. ^**^ Random human SNPs were generated using a python library from the Single Nucleotide Polymorphism database (dbSNP build 151; November 10, 2017). A total of 80 randomly generated data sets of 180 SNPs were run through the CoDeS3D algorithm. SEM, standard error of the mean; SNP, single nucleotide polymorphism; eQTL, expression quantitative trait loci; eGene, a gene whose expression is associated with an eQTL.

**Supplementary Table 2. Spatial interactions between T1D-associated eQTLs and genes**

| *SNP  (T1D-eQTLs) | SNP Chr. | SNP Locus | **Gene Name | Gene Chr. | Gene Start | Gene End | SNP-gene Distance | Cell Lines |
| --- | --- | --- | --- | --- | --- | --- | --- | --- |
| rs2358994 | 1 | 114429460 | **AP4B1-AS1** | 1 | 114399257 | 114443859 | 0 | KBM7 |
| rs2153977 | 1 | 114080070 | **MAGI3** | 1 | 113933371 | 114228545 | 0 | KBM7 |
| rs3024505 | 1 | 206939903 | **PLXNA2** | 1 | 208195587 | 208417665 | 1255684 | HUVEC |
| rs3024505 | 1 | 206939903 | **RAB3GAP2** | 1 | 220321635 | 220445796 | 13381732 | K562 |
| rs2153977 | 1 | 114080070 | **TSPAN2** | 1 | 115590632 | 115632121 | 1510562 | KBM7 |
| rs2358994 | 1 | 114429460 | **MAGI3** | 1 | 113933371 | 114228545 | 200915 | IMR90 |
| rs2153977 | 1 | 114080070 | **PHTF1** | 1 | 114239453 | 114302111 | 159383 | NHEK |
| rs1983853 | 1 | 85311191 | **ZNF644** | 1 | 91380859 | 91487829 | 6069668 | NHEK |
| rs2153977 | 1 | 114080070 | **SLC6A17** | 1 | 110693108 | 110744824 | 3335246 | HUVEC |
| rs2476601 | 1 | 114377567 | **AP4B1-AS1** | 1 | 114399257 | 114443859 | 21690 | KBM7 |
| rs6679677 | 1 | 114303807 | **AP4B1-AS1** | 1 | 114399257 | 114443859 | 95450 | HUVEC |
| rs11580078 | 1 | 67669633 | **HHAT** | 1 | 210501596 | 210849638 | 142831963 | K562 |
| rs2990510 | 1 | 197020657 | **F13B** | 1 | 197008321 | 197036397 | 0 | KBM7 |
| rs2269241 | 1 | 64108770 | **PGM1** | 1 | 64058947 | 64125916 | 0 | KBM7 |
| rs2476601 | 1 | 114377567 | **PHTF1** | 1 | 114239453 | 114302111 | 75456 | NHEK |
| rs478222 | 2 | 25301754 | **ADCY3** | 2 | 25042038 | 25142708 | 159046 | KBM7 |
| rs36001488 | 2 | 234185266 | **ATG16L1** | 2 | 234118697 | 234204320 | 0 | KBM7 |
| rs1990760 | 2 | 163124050 | **TXNL4B** | 16 | 72078188 | 72128330 | NA | KBM7 |
| rs478222 | 2 | 25301754 | **RP11-509E16.1** | 2 | 25380063 | 25382006 | 78309 | KBM7 |
| rs10186458 | 2 | 20598509 | **SDC1** | 2 | 20400558 | 20425194 | 173315 | NHEK |
| rs9653442 | 2 | 100825366 | **AFF3** | 2 | 100162323 | 100759201 | 66165 | KBM7 |
| rs2165738 | 2 | 24692808 | **ADCY3** | 2 | 25042038 | 25142708 | 349230 | KBM7 |
| rs836589 | 2 | 173493077 | **PDK1** | 2 | 173420101 | 173489823 | 3254 | KBM7 |
| rs6547853 | 2 | 28646800 | **RP11-373D23.3** | 2 | 28648812 | 28649586 | 2012 | IMR90 |
| rs478222 | 2 | 25301754 | **DNAJC27-AS1** | 2 | 25194259 | 25262563 | 39191 | KBM7 |
| rs836589 | 2 | 173493077 | **RAPGEF4-AS1** | 2 | 173541869 | 173600934 | 48792 | KBM7 |
| rs478222 | 2 | 25301754 | **RBMS3** | 3 | 29322473 | 30051886 | NA | HUVEC |
| rs478222 | 2 | 25301754 | **NCOA1** | 2 | 24714783 | 24993571 | 308183 | KBM7 |
| rs36001488 | 2 | 234185266 | **USP40** | 2 | 234384166 | 234475428 | 198900 | KBM7 |
| rs4676410 | 2 | 241563738 | **GPR35** | 2 | 241544848 | 241570676 | 0 | KBM7 |
| rs9653442 | 2 | 100825366 | **SULT1C2** | 2 | 108905095 | 108926371 | 8079729 | IMR90 |
| rs3087243 | 2 | 204738918 | **CTLA4** | 2 | 204732509 | 204738683 | 235 | KBM7 |
| rs478222 | 2 | 25301754 | **SLC8A1** | 2 | 40324410 | 40838193 | 15022656 | KBM7 |
| rs7588550 | 2 | 213168767 | **ERBB4** | 2 | 212240446 | 213403565 | 0 | KBM7 |
| rs9653442 | 2 | 100825366 | **LONRF2** | 2 | 100889753 | 100939195 | 64387 | IMR90 |
| rs2165738 | 2 | 24692808 | **DNAJC27** | 2 | 25166505 | 25194963 | 473697 | HUVEC |
| rs478222 | 2 | 25301754 | **DNAJC27** | 2 | 25166505 | 25194963 | 106791 | KBM7 |
| rs4625 | 3 | 49572139 | **AMT** | 3 | 49454211 | 49460186 | 111953 | KBM7 |
| rs4625 | 3 | 49572139 | **APEH** | 3 | 49711435 | 49721396 | 139296 | KBM7 |
| rs4625 | 3 | 49572139 | **QRICH1** | 3 | 49067140 | 49131796 | 440343 | KBM7 |
| rs4625 | 3 | 49572139 | **RBM6** | 3 | 49977440 | 50137478 | 405301 | KBM7 |
| rs4625 | 3 | 49572139 | **RNF123** | 3 | 49726932 | 49758962 | 154793 | KBM7 |
| rs4625 | 3 | 49572139 | **DOCK3** | 3 | 50712672 | 51421629 | 1140533 | NHEK |
| rs4625 | 3 | 49572139 | **DAG1** | 3 | 49506146 | 49573048 | 0 | KBM7 |
| rs4625 | 3 | 49572139 | **NPAT** | 11 | 108027942 | 108093369 | NA | K562 |
| rs9850225 | 3 | 80865763 | **PRICKLE2** | 3 | 64079543 | 64431152 | 16434611 | KBM7 |
| rs9850225 | 3 | 80865763 | **RP11-47P18.2** | 3 | 80814048 | 80838505 | 27258 | NHEK |
| rs4625 | 3 | 49572139 | **ACLY** | 17 | 40023161 | 40086795 | NA | HUVEC |
| rs71597855 | 4 | 54656436 | **UNC5C** | 4 | 96083655 | 96470357 | 41427219 | NHEK |
| rs10517086 | 4 | 26085510 | **RBPJ** | 4 | 26165077 | 26436541 | 79567 | KBM7 |
| rs17388568 | 4 | 123329361 | **KIAA1109** | 4 | 123073488 | 123283913 | 45448 | NHEK |
| rs7672495 | 4 | 4992366 | **EVC** | 4 | 5712924 | 5830772 | 720558 | KBM7 |
| rs4505848 | 4 | 123132491 | **KIAA1109** | 4 | 123073488 | 123283913 | 0 | KBM7 |
| rs4505848 | 4 | 123132491 | **ELF2** | 4 | 139949266 | 140098372 | 16816775 | NHEK |
| rs317865 | 4 | 16156665 | **ZEB2P1** | 4 | 16362309 | 16398946 | 205644 | HUVEC |
| rs7672495 | 4 | 4992366 | **MEF2C-AS1** | 5 | 88179145 | 88762215 | NA | HUVEC |
| rs7660520 | 4 | 183745320 | **SORBS2** | 4 | 186506598 | 186877806 | 2761278 | NHEK |
| rs317865 | 4 | 16156665 | **TAPT1-AS1** | 4 | 16228286 | 16321763 | 71621 | KBM7 |
| rs11741255 | 5 | 131811181 | **AC034220.3** | 5 | 131646978 | 131705608 | 105573 | NHEK |
| rs4869313 | 5 | 96223879 | **ERAP2** | 5 | 96211643 | 96255420 | 0 | KBM7 |
| rs4869313 | 5 | 96223879 | **LNPEP** | 5 | 96271098 | 96373219 | 47219 | NHEK |
| rs11741255 | 5 | 131811181 | **SLC22A5** | 5 | 131705444 | 131731306 | 79875 | IMR90 |
| rs11741255 | 5 | 131811181 | **P4HA2** | 5 | 131527531 | 131631008 | 180173 | IMR90 |
| rs4869313 | 5 | 96223879 | **ERAP1** | 5 | 96096521 | 96143803 | 80076 | IMR90 |
| rs17166496 | 5 | 132628883 | **UBAC2** | 13 | 99853028 | 100038688 | NA | NHEK |
| rs11741255 | 5 | 131811181 | **AC116366.6** | 5 | 131804582 | 131808735 | 2446 | NHEK |
| rs7731626 | 5 | 55444682 | **ANKRD55** | 5 | 55395507 | 55529186 | 0 | KBM7 |
| rs886424 | 6 | 30782001 | **BTN3A2** | 6 | 26365387 | 26378546 | 4403455 | KBM7 |
| rs9268645 | 6 | 32408526 | **ATF6B** | 6 | 32065953 | 32096030 | 312496 | KBM7 |
| rs1270942 | 6 | 31918859 | **CCHCR1** | 6 | 31110216 | 31126015 | 792844 | HUVEC |
| rs9388489 | 6 | 126698718 | **CENPW** | 6 | 126661320 | 126670021 | 28697 | NHEK |
| rs1270942 | 6 | 31918859 | **CLIC1** | 6 | 31698358 | 31707540 | 211319 | KBM7 |
| rs886424 | 6 | 30782001 | **FLOT1** | 6 | 30695486 | 30710510 | 71491 | KBM7 |
| rs9268645 | 6 | 32408526 | **HLA-DQA2** | 6 | 32709119 | 32714992 | 300593 | HUVEC |
| rs9268645 | 6 | 32408526 | **HLA-DQB1** | 6 | 32627244 | 32636160 | 218718 | KBM7 |
| rs1270942 | 6 | 31918859 | **HLA-DQB2** | 6 | 32723875 | 32731311 | 805016 | HUVEC |
| rs9268645 | 6 | 32408526 | **HLA-DQB2** | 6 | 32723875 | 32731311 | 315349 | IMR90 |
| rs9268645 | 6 | 32408526 | **HLA-DRB1** | 6 | 32546546 | 32557625 | 138020 | IMR90 |
| rs2251396 | 6 | 31364706 | **MICA** | 6 | 31371356 | 31383092 | 6650 | NHEK |
| rs1980493 | 6 | 32363214 | **NOTCH4** | 6 | 32162620 | 32191844 | 171370 | KBM7 |
| rs2251396 | 6 | 31364706 | **PSORS1C1** | 6 | 31082527 | 31107869 | 256837 | KBM7 |
| rs1270942 | 6 | 31918859 | **SKIV2L** | 6 | 31926857 | 31937532 | 7998 | KBM7 |
| rs1015166 | 6 | 32798730 | **TAP2** | 6 | 32781544 | 32806599 | 0 | KBM7 |
| rs9268645 | 6 | 32408526 | **HLA-DQB1-AS1** | 6 | 32627657 | 32628506 | 219131 | KBM7 |
| rs1980493 | 6 | 32363214 | **HLA-DQB2** | 6 | 32723875 | 32731311 | 360661 | KBM7 |
| rs1270942 | 6 | 31918859 | **PSORS1C1** | 6 | 31082527 | 31107869 | 810990 | KBM7 |
| rs1270942 | 6 | 31918859 | **DXO** | 6 | 31937587 | 31940069 | 18728 | IMR90 |
| rs1270942 | 6 | 31918859 | **RNF5** | 6 | 32146131 | 32151930 | 227272 | IMR90 |
| rs1270942 | 6 | 31918859 | **PPP1R18** | 6 | 30644166 | 30655672 | 1263187 | K562 |
| rs2857595 | 6 | 31568468 | **MSH5** | 6 | 31707725 | 31732622 | 139257 | NHEK |
| rs1270942 | 6 | 31918859 | **MSH5** | 6 | 31707725 | 31732622 | 186237 | K562 |
| rs886424 | 6 | 30782001 | **PPP1R18** | 6 | 30644166 | 30655672 | 126329 | IMR90 |
| rs2523989 | 6 | 30078274 | **ZNRD1-AS1** | 6 | 29968788 | 30029417 | 48857 | KBM7 |
| rs1270942 | 6 | 31918859 | **WDR7** | 18 | 54318574 | 54698828 | NA | HUVEC |
| rs1980493 | 6 | 32363214 | **HLA-DMA** | 6 | 32916390 | 32936871 | 553176 | K562 |
| rs1015166 | 6 | 32798730 | **HLA-DMA** | 6 | 32916390 | 32936871 | 117660 | HUVEC |
| rs1015166 | 6 | 32798730 | **HLA-DOB** | 6 | 32780540 | 32784825 | 13905 | K562 |
| rs1980493 | 6 | 32363214 | **HLA-DQA2** | 6 | 32709119 | 32714992 | 345905 | NHEK |
| rs3761980 | 6 | 35993905 | **MAPK14** | 6 | 35995488 | 36079013 | 1583 | NHEK |
| rs2857595 | 6 | 31568468 | **SAMD12** | 8 | 119201698 | 119634234 | NA | HUVEC |
| rs13217044 | 6 | 8226996 | **GALNT11** | 7 | 151722759 | 151819425 | NA | K562 |
| rs2251396 | 6 | 31364706 | **MICB** | 6 | 31462658 | 31478901 | 97952 | K562 |
| rs2857595 | 6 | 31568468 | **MICB** | 6 | 31462658 | 31478901 | 89567 | IMR90 |
| rs1270942 | 6 | 31918859 | **FLRT3** | 20 | 14303634 | 14318262 | NA | NHEK |
| rs886424 | 6 | 30782001 | **HCG17** | 6 | 30201816 | 30293911 | 488090 | KBM7 |
| rs1270942 | 6 | 31918859 | **HLA-DMB** | 6 | 32902406 | 32908847 | 983547 | K562 |
| rs2523989 | 6 | 30078274 | **VARS2** | 6 | 30876019 | 30894236 | 797745 | KBM7 |
| rs1015166 | 6 | 32798730 | **RPS5** | 19 | 58897767 | 58906173 | NA | KBM7 |
| rs9268645 | 6 | 32408526 | **NOTCH4** | 6 | 32162620 | 32191844 | 216682 | K562 |
| rs886424 | 6 | 30782001 | **HCG20** | 6 | 30734602 | 30760027 | 21974 | KBM7 |
| rs6931865 | 6 | 144079853 | **PHACTR2** | 6 | 143857982 | 144152322 | 0 | KBM7 |
| rs2251396 | 6 | 31364706 | **CCHCR1** | 6 | 31110216 | 31126015 | 238691 | KBM7 |
| rs886424 | 6 | 30782001 | **IER3** | 6 | 30710976 | 30712331 | 69670 | NHEK |
| rs886424 | 6 | 30782001 | **LINC00243** | 6 | 30766431 | 30798436 | 0 | KBM7 |
| rs924043 | 6 | 170379024 | **HLA-DPA1** | 6 | 33032346 | 33048552 | 137330472 | KBM7 |
| rs1980493 | 6 | 32363214 | **HLA-DOB** | 6 | 32780540 | 32784825 | 417326 | NHEK |
| rs9388489 | 6 | 126698718 | **UBAC1** | 9 | 138824815 | 138853226 | NA | HUVEC |
| rs1980493 | 6 | 32363214 | **ATF6B** | 6 | 32065953 | 32096030 | 267184 | IMR90 |
| rs1980493 | 6 | 32363214 | **GPANK1** | 6 | 31629006 | 31634060 | 729154 | KBM7 |
| rs3761980 | 6 | 35993905 | **RP1-179N16.6** | 6 | 36114475 | 36164982 | 120570 | IMR90 |
| rs1270942 | 6 | 31918859 | **AGER** | 6 | 32148745 | 32152101 | 229886 | IMR90 |
| rs2251396 | 6 | 31364706 | **JMJD1C** | 10 | 64926981 | 65225722 | NA | HUVEC |
| rs886424 | 6 | 30782001 | **RNF39** | 6 | 30038043 | 30043664 | 738337 | KBM7 |
| rs886424 | 6 | 30782001 | **HCP5** | 6 | 31368479 | 31445283 | 586478 | KBM7 |
| rs2251396 | 6 | 31364706 | **HLA-DQB2** | 6 | 32723875 | 32731311 | 1359169 | KBM7 |
| rs1270942 | 6 | 31918859 | **HCP5** | 6 | 31368479 | 31445283 | 473576 | KBM7 |
| rs2251396 | 6 | 31364706 | **NOTCH4** | 6 | 32162620 | 32191844 | 797914 | KBM7 |
| rs1980493 | 6 | 32363214 | **HLA-DQB1** | 6 | 32627244 | 32636160 | 264030 | KBM7 |
| rs3761980 | 6 | 35993905 | **SRPK1** | 6 | 35800743 | 35889119 | 104786 | KBM7 |
| rs2523989 | 6 | 30078274 | **TRIM26** | 6 | 30152232 | 30181204 | 73958 | KBM7 |
| rs886424 | 6 | 30782001 | **GRM4** | 6 | 33989628 | 34123399 | 3207627 | KBM7 |
| rs1270942 | 6 | 31918859 | **C2** | 6 | 31865562 | 31913449 | 5410 | KBM7 |
| rs1980493 | 6 | 32363214 | **C2** | 6 | 31865562 | 31913449 | 449765 | NHEK |
| rs886424 | 6 | 30782001 | **HLA-L** | 6 | 30227361 | 30260791 | 521210 | KBM7 |
| rs7804356 | 7 | 26891664 | **HOXA5** | 7 | 27180671 | 27183287 | 289007 | KBM7 |
| rs7804356 | 7 | 26891664 | **SKAP2** | 7 | 26706681 | 27034858 | 0 | KBM7 |
| rs550448 | 7 | 28229041 | **AACS** | 12 | 125549925 | 125627873 | NA | NHEK |
| rs12679857 | 8 | 119977336 | **UBE2E3** | 2 | 181831975 | 181941312 | NA | NHEK |
| rs2410601 | 8 | 18922576 | **PSD3** | 8 | 18384811 | 18942240 | 0 | KBM7 |
| rs2410601 | 8 | 18922576 | **RP11-420B22.1** | 8 | 18846789 | 18858462 | 64114 | KBM7 |
| rs657152 | 9 | 136139264 | **ABO** | 9 | 136125788 | 136150617 | 0 | KBM7 |
| rs579459 | 9 | 136154167 | **ABO** | 9 | 136125788 | 136150617 | 3550 | KBM7 |
| rs4246905 | 9 | 117553248 | **CENPP** | 9 | 95087766 | 95382815 | 22170433 | NHEK |
| rs10988542 | 9 | 132657263 | **USP20** | 9 | 132596977 | 132644107 | 13156 | NHEK |
| rs11145763 | 9 | 139263595 | **SNAPC4** | 9 | 139270029 | 139293249 | 6434 | HUVEC |
| rs4246905 | 9 | 117553248 | **TNFSF15** | 9 | 117546915 | 117568406 | 0 | KBM7 |
| rs7100025 | 10 | 37592537 | **ZNF25** | 10 | 38238500 | 38265561 | 645963 | NHEK |
| rs1332099 | 10 | 101298450 | **RP11-129J12.1** | 10 | 101286838 | 101288018 | 10432 | NHEK |
| rs10822050 | 10 | 64438770 | **ADO** | 10 | 64564516 | 64568238 | 125746 | HUVEC |
| rs1326934 | 10 | 97284080 | **SORBS1** | 10 | 97071528 | 97321171 | 0 | KBM7 |
| rs10509540 | 10 | 90023032 | **PTEN** | 10 | 89622870 | 89731687 | 291345 | KBM7 |
| rs10786436 | 10 | 100300181 | **HPSE2** | 10 | 100218875 | 100995619 | 0 | KBM7 |
| rs10822050 | 10 | 64438770 | **CNTN1** | 12 | 41086244 | 41466220 | NA | KBM7 |
| rs7100025 | 10 | 37592537 | **ATP8A2P1** | 10 | 37537046 | 37604729 | 0 | KBM7 |
| rs17885785 | 11 | 2167849 | **IGF2-AS** | 11 | 2161731 | 2169894 | 0 | KBM7 |
| rs1004446 | 11 | 2170142 | **IGF2-AS** | 11 | 2161731 | 2169894 | 248 | KBM7 |
| rs689 | 11 | 2182223 | **IGF2-AS** | 11 | 2161731 | 2169894 | 12329 | KBM7 |
| rs7111341 | 11 | 2213165 | **TMEM80** | 11 | 695428 | 705028 | 1508137 | NHEK |
| rs1004446 | 11 | 2170142 | **IGF2** | 11 | 2150342 | 2170833 | 0 | KBM7 |
| rs3741208 | 11 | 2169773 | **IGF2-AS** | 11 | 2161731 | 2169894 | 0 | KBM7 |
| rs3741208 | 11 | 2169773 | **EIF3F** | 11 | 7991798 | 8023409 | 5822025 | NHEK |
| rs4084127 | 11 | 69596522 | **SUV420H1** | 11 | 67922330 | 67981295 | 1615227 | K562 |
| rs1701704 | 12 | 56412486 | **PDE1B** | 12 | 54943134 | 54973023 | 1439463 | KBM7 |
| rs3764021 | 12 | 9833627 | **CLECL1** | 12 | 9868456 | 9885895 | 34829 | KBM7 |
| rs11052552 | 12 | 9855957 | **CLECL1** | 12 | 9868456 | 9885895 | 12499 | NHEK |
| rs4763879 | 12 | 9910163 | **CLECL1** | 12 | 9868456 | 9885895 | 24268 | KBM7 |
| rs3764021 | 12 | 9833627 | **RP11-705C15.2** | 12 | 9787188 | 9811008 | 22619 | KBM7 |
| rs11052552 | 12 | 9855957 | **RP11-705C15.2** | 12 | 9787188 | 9811008 | 44949 | NHEK |
| rs3764021 | 12 | 9833627 | **RP11-75L1.2** | 12 | 9847980 | 9848823 | 14353 | KBM7 |
| rs1701704 | 12 | 56412486 | **RARG** | 12 | 53604354 | 53626764 | 2785722 | HUVEC |
| rs1689510 | 12 | 56396767 | **SUOX** | 12 | 56390964 | 56400425 | 0 | NHEK |
| rs614226 | 12 | 120974509 | **POP5** | 12 | 121016567 | 121019201 | 42058 | HUVEC |
| rs1701704 | 12 | 56412486 | **SUOX** | 12 | 56390964 | 56400425 | 12061 | KBM7 |
| rs2292239 | 12 | 56482179 | **SUOX** | 12 | 56390964 | 56400425 | 81754 | IMR90 |
| rs614226 | 12 | 120974509 | **DYNLL1-AS1** | 12 | 120928131 | 120933743 | 40766 | NHEK |
| rs614226 | 12 | 120974509 | **RNF10** | 12 | 120971283 | 121015397 | 0 | KBM7 |
| rs614226 | 12 | 120974509 | **DYNLL1** | 12 | 120907653 | 120936296 | 38213 | KBM7 |
| rs614226 | 12 | 120974509 | **CCDC92** | 12 | 124403207 | 124457378 | 3428698 | NHEK |
| rs3184504 | 12 | 111884607 | **ARHGAP42** | 11 | 100558384 | 100862668 | NA | HUVEC |
| rs1701704 | 12 | 56412486 | **IKZF4** | 12 | 56401443 | 56432219 | 0 | KBM7 |
| rs11171739 | 12 | 56470624 | **PDE4DIP** | 1 | 144836157 | 145076186 | NA | K562 |
| rs17696736 | 12 | 112486817 | **ALDH2** | 12 | 112204691 | 112247782 | 239035 | K562 |
| rs2292239 | 12 | 56482179 | **IKZF4** | 12 | 56401443 | 56432219 | 49960 | HUVEC |
| rs614226 | 12 | 120974509 | **COQ5** | 12 | 120941077 | 120972237 | 2272 | KBM7 |
| rs3764021 | 12 | 9833627 | **CLEC2B** | 12 | 10005583 | 10022735 | 171956 | HUVEC |
| rs1701704 | 12 | 56412486 | **RAB5B** | 12 | 56367697 | 56388490 | 23996 | NHEK |
| rs2292239 | 12 | 56482179 | **RAB5B** | 12 | 56367697 | 56388490 | 93689 | IMR90 |
| rs2292239 | 12 | 56482179 | **RELT** | 11 | 73087309 | 73108519 | NA | IMR90 |
| rs17466626 | 12 | 40760630 | **LRRK2** | 12 | 40590546 | 40763087 | 0 | KBM7 |
| rs3764021 | 12 | 9833627 | **RP11-705C15.3** | 12 | 9811163 | 9814681 | 18946 | KBM7 |
| rs614226 | 12 | 120974509 | **HLA-E** | 6 | 30457244 | 30461982 | NA | IMR90 |
| rs614226 | 12 | 120974509 | **MSI1** | 12 | 120779133 | 120806983 | 167526 | KBM7 |
| rs17466626 | 12 | 40760630 | **LEMD3** | 12 | 65563351 | 65642107 | 24802721 | NHEK |
| rs3764021 | 12 | 9833627 | **KLRB1** | 12 | 9747147 | 9760482 | 73145 | KBM7 |
| rs11052552 | 12 | 9855957 | **KLRB1** | 12 | 9747147 | 9760482 | 95475 | HUVEC |
| rs4763879 | 12 | 9910163 | **KLRF1** | 12 | 9980077 | 9997606 | 69914 | NHEK |
| rs539514 | 13 | 76326281 | **LMO7** | 13 | 76194570 | 76434004 | 0 | KBM7 |
| rs10137082 | 14 | 23840032 | **EFS** | 14 | 23825611 | 23834961 | 5071 | KBM7 |
| rs3825932 | 15 | 79235445 | **CTSH** | 15 | 79213400 | 79241916 | 0 | KBM7 |
| rs3825932 | 15 | 79235445 | **ADAMTS7** | 15 | 79051545 | 79103773 | 131672 | KBM7 |
| rs7171171 | 15 | 38907040 | **RASGRP1** | 15 | 38780304 | 38857776 | 49264 | KBM7 |
| rs72743477 | 15 | 67464290 | **AAGAB** | 15 | 67493371 | 67547533 | 29081 | KBM7 |
| rs8035957 | 15 | 38838263 | **RASGRP1** | 15 | 38780304 | 38857776 | 0 | KBM7 |
| rs8035957 | 15 | 38838263 | **RP11-102L12.2** | 15 | 38795054 | 38797182 | 41081 | NHEK |
| rs72743477 | 15 | 67464290 | **SMAD3** | 15 | 67356101 | 67487533 | 0 | KBM7 |
| rs72743477 | 15 | 67464290 | **TCF12** | 15 | 57210821 | 57591479 | 9872811 | HUVEC |
| rs12928404 | 16 | 28847245 | **ATXN2L** | 16 | 28834356 | 28848558 | 0 | KBM7 |
| rs7202877 | 16 | 75247244 | **CFDP1** | 16 | 75327596 | 75467383 | 80352 | NHEK |
| rs12928404 | 16 | 28847245 | **NFATC2IP** | 16 | 28962128 | 28978418 | 114883 | KBM7 |
| rs416603 | 16 | 11364078 | **RMI2** | 16 | 11343476 | 11445619 | 0 | KBM7 |
| rs12928404 | 16 | 28847245 | **SH2B1** | 16 | 28857921 | 28885526 | 10676 | NHEK |
| rs12928404 | 16 | 28847245 | **TUFM** | 16 | 28853732 | 28857729 | 6487 | KBM7 |
| rs4788084 | 16 | 28539847 | **RABEP2** | 16 | 28915742 | 28947847 | 375895 | HUVEC |
| rs12928404 | 16 | 28847245 | **RABEP2** | 16 | 28915742 | 28947847 | 68497 | KBM7 |
| rs4788084 | 16 | 28539847 | **SBK1** | 16 | 28303840 | 28335170 | 204677 | HUVEC |
| rs12928404 | 16 | 28847245 | **SULT1A1** | 16 | 28616903 | 28634946 | 212299 | KBM7 |
| rs12598357 | 16 | 28340944 | **CCDC101** | 16 | 28565236 | 28603111 | 224292 | K562 |
| rs12928404 | 16 | 28847245 | **LAT** | 16 | 28996147 | 29002104 | 148902 | HUVEC |
| rs12708716 | 16 | 11179872 | **MAP3K9** | 14 | 71189243 | 71276251 | NA | KBM7 |
| rs77150043 | 16 | 50304248 | **SSR2** | 1 | 155978839 | 155990750 | NA | KBM7 |
| rs2903692 | 16 | 11238782 | **LINC00535** | 8 | 94225531 | 94712661 | NA | KBM7 |
| rs12928404 | 16 | 28847245 | **ATP2A1** | 16 | 28889726 | 28915830 | 42481 | KBM7 |
| rs12708716 | 16 | 11179872 | **NUP93** | 16 | 56764017 | 56878797 | 45584145 | K562 |
| rs12928404 | 16 | 28847245 | **APOBR** | 16 | 28505970 | 28510291 | 336954 | KBM7 |
| rs7202877 | 16 | 75247244 | **TMEM170A** | 16 | 75476952 | 75499395 | 229708 | IMR90 |
| rs12708716 | 16 | 11179872 | **RMI2** | 16 | 11343476 | 11445619 | 163604 | KBM7 |
| rs2903692 | 16 | 11238782 | **RMI2** | 16 | 11343476 | 11445619 | 104694 | KBM7 |
| rs7202877 | 16 | 75247244 | **BCAR1** | 16 | 75262928 | 75301951 | 15684 | KBM7 |
| rs77150043 | 16 | 50304248 | **BRD7** | 16 | 50347398 | 50402845 | 43150 | KBM7 |
| rs12928404 | 16 | 28847245 | **CCDC101** | 16 | 28565236 | 28603111 | 244134 | NHEK |
| rs12232497 | 17 | 38040118 | **GSDMA** | 17 | 38119226 | 38134019 | 79108 | NHEK |
| rs2872507 | 17 | 38040762 | **GSDMA** | 17 | 38119226 | 38134019 | 78464 | IMR90 |
| rs12232497 | 17 | 38040118 | **ORMDL3** | 17 | 38077294 | 38083854 | 37176 | HUVEC |
| rs2872507 | 17 | 38040762 | **ORMDL3** | 17 | 38077294 | 38083854 | 36532 | NHEK |
| rs2290400 | 17 | 38066239 | **ORMDL3** | 17 | 38077294 | 38083854 | 11055 | NHEK |
| rs7221109 | 17 | 38770285 | **SMARCE1** | 17 | 38781214 | 38804760 | 10929 | IMR90 |
| rs2290400 | 17 | 38066239 | **PGAP3** | 17 | 37827375 | 37853050 | 213189 | NHEK |
| rs12232497 | 17 | 38040118 | **MED24** | 17 | 38175350 | 38217468 | 135232 | KBM7 |
| rs2872507 | 17 | 38040762 | **MED24** | 17 | 38175350 | 38217468 | 134588 | KBM7 |
| rs2290400 | 17 | 38066239 | **GSDMB** | 17 | 38060848 | 38076107 | 0 | KBM7 |
| rs7221109 | 17 | 38770285 | **VSX1** | 20 | 25051521 | 25062996 | NA | KBM7 |
| rs7221109 | 17 | 38770285 | **CCR7** | 17 | 38710021 | 38721724 | 48561 | KBM7 |
| rs12232497 | 17 | 38040118 | **GSDMB** | 17 | 38060848 | 38076107 | 20730 | KBM7 |
| rs2872507 | 17 | 38040762 | **GSDMB** | 17 | 38060848 | 38076107 | 20086 | KBM7 |
| rs2872507 | 17 | 38040762 | **RP11-94L15.2** | 17 | 37913974 | 37920089 | 120673 | HUVEC |
| rs2872507 | 17 | 38040762 | **PSMD3** | 17 | 38137050 | 38154213 | 96288 | NHEK |
| rs2290400 | 17 | 38066239 | **PSMD3** | 17 | 38137050 | 38154213 | 70811 | NHEK |
| rs2290400 | 17 | 38066239 | **STARD3** | 17 | 37793318 | 37819737 | 246502 | NHEK |
| rs2872507 | 17 | 38040762 | **ERBB2** | 17 | 37844167 | 37886679 | 154083 | HUVEC |
| rs12232497 | 17 | 38040118 | **PGAP3** | 17 | 37827375 | 37853050 | 187068 | NHEK |
| rs2290400 | 17 | 38066239 | **MSL1** | 17 | 38278551 | 38293042 | 212312 | IMR90 |
| rs2290400 | 17 | 38066239 | **ERBB2** | 17 | 37844167 | 37886679 | 179560 | NHEK |
| rs2872507 | 17 | 38040762 | **CLSTN2** | 3 | 139654027 | 140296239 | NA | KBM7 |
| rs12232497 | 17 | 38040118 | **ZPBP2** | 17 | 38024417 | 38034149 | 5969 | KBM7 |
| rs2872507 | 17 | 38040762 | **ZPBP2** | 17 | 38024417 | 38034149 | 6613 | K562 |
| rs2290400 | 17 | 38066239 | **ZPBP2** | 17 | 38024417 | 38034149 | 32090 | KBM7 |
| rs16956936 | 17 | 7633691 | **KCNAB3** | 17 | 7825177 | 7833121 | 191486 | NHEK |
| rs2542151 | 18 | 12779946 | **THSD4** | 15 | 71389291 | 72075722 | NA | KBM7 |
| rs1893217 | 18 | 12809339 | **DSCAM** | 21 | 41382926 | 42219065 | NA | HUVEC |
| rs763361 | 18 | 67531641 | **CD226** | 18 | 67498394 | 67629039 | 0 | KBM7 |
| rs763361 | 18 | 67531641 | **DOK6** | 18 | 67068291 | 67516323 | 15318 | KBM7 |
| rs2542151 | 18 | 12779946 | **JUP** | 17 | 39775692 | 39943183 | NA | KBM7 |
| rs425105 | 19 | 47208480 | **PRKD2** | 19 | 47177532 | 47220384 | 0 | KBM7 |
| rs2304256 | 19 | 10475651 | **TYK2** | 19 | 10461209 | 10491352 | 0 | KBM7 |
| rs602662 | 19 | 49206984 | **IZUMO1** | 19 | 49244109 | 49250166 | 37125 | KBM7 |
| rs602662 | 19 | 49206984 | **MAMSTR** | 19 | 49215999 | 49222978 | 9015 | KBM7 |
| rs602662 | 19 | 49206984 | **FUT2** | 19 | 49199228 | 49209207 | 0 | KBM7 |
| rs2304256 | 19 | 10475651 | **CHAF1A** | 19 | 4402659 | 4445015 | 6030636 | K562 |
| rs2304256 | 19 | 10475651 | **MRPL4** | 19 | 10362577 | 10370721 | 104930 | KBM7 |
| rs602662 | 19 | 49206984 | **RASIP1** | 19 | 49223844 | 49243978 | 16860 | KBM7 |
| rs425105 | 19 | 47208480 | **SLC1A5** | 19 | 47278140 | 47291851 | 69660 | NHEK |
| rs2304256 | 19 | 10475651 | **CDC37** | 19 | 10501810 | 10530797 | 26159 | KBM7 |
| rs2304256 | 19 | 10475651 | **ICAM5** | 19 | 10400657 | 10407454 | 68197 | KBM7 |
| rs602662 | 19 | 49206984 | **CAMTA1** | 1 | 6845384 | 7829766 | NA | K562 |
| rs425105 | 19 | 47208480 | **STRN4** | 19 | 47222764 | 47250251 | 14284 | KBM7 |
| rs11698685 | 20 | 58895845 | **SLC1A3** | 5 | 36606457 | 36688436 | NA | K562 |
| rs2281808 | 20 | 1610550 | **PRAM1** | 19 | 8554940 | 8567996 | NA | KBM7 |
| rs2281808 | 20 | 1610550 | **RP11-77C3.3** | 20 | 1614154 | 1629118 | 3604 | NHEK |
| rs11203203 | 21 | 43836185 | **TIAM1** | 21 | 32490734 | 32932290 | 10903895 | IMR90 |
| rs2836882 | 21 | 40466569 | **AP3B1** | 5 | 77296349 | 77590579 | NA | HUVEC |
| rs9976767 | 21 | 43836389 | **SYNGAP1** | 6 | 33387847 | 33421466 | NA | K562 |
| rs3788013 | 21 | 43841327 | **SLC37A1** | 21 | 43916118 | 44001550 | 74791 | KBM7 |
| rs2836882 | 21 | 40466569 | **ETS2** | 21 | 40177231 | 40196879 | 269690 | IMR90 |
| rs3788013 | 21 | 43841327 | **GNAO1** | 16 | 56225302 | 56391356 | NA | K562 |
| rs2836882 | 21 | 40466569 | **AF064858.8** | 21 | 40360633 | 40378079 | 88490 | KBM7 |
| rs5753037 | 22 | 30581721 | **MTMR3** | 22 | 30279144 | 30426855 | 154866 | KBM7 |
| rs743777 | 22 | 37551606 | **SMCHD1** | 18 | 2655737 | 2805015 | NA | KBM7 |
| rs229541 | 22 | 37591317 | **C1QTNF6** | 22 | 37576207 | 37595425 | 0 | KBM7 |
| rs5753037 | 22 | 30581721 | **ZMAT5** | 22 | 30126945 | 30163000 | 418721 | KBM7 |
| rs5753037 | 22 | 30581721 | **ASCC2** | 22 | 30184597 | 30234271 | 347450 | KBM7 |
| rs229541 | 22 | 37591317 | **RAC2** | 22 | 37621301 | 37640488 | 29984 | KBM7 |
| rs2664170 | X | 153945601 | **F8** | X | 154064063 | 154255215 | 118462 | KBM7 |
| rs2664170 | X | 153945601 | **TMLHE** | X | 154719776 | 154899605 | 774175 | NHEK |
| rs2664170 | X | 153945601 | **FUNDC2** | X | 154254255 | 154288578 | 308654 | IMR90 |
| rs2664170 | X | 153945601 | **BRCC3** | X | 154299695 | 154351349 | 354094 | KBM7 |
| rs2664170 | X | 153945601 | **PLXNA3** | X | 153686621 | 153701989 | 243612 | IMR90 |
| rs2664170 | X | 153945601 | **RAB39B** | X | 154487526 | 154493874 | 541925 | K562 |
| rs2664170 | X | 153945601 | **THOC7** | 3 | 63819546 | 63849579 | NA | KBM7 |

Spatial interactions between T1D-associated eQTLs and genes. T1D-associated SNPs were identified in the GWAS catalogue. Non-redundant interactions between SNPs* and genes** are analyses from the computational algorithm (CoDeS3D). Cis-interactions were defined as occurring in loci <1Mb apart, and trans-interactions were defined as occurring between loci >1Mb apart, or on different chromosomes (FDR ≤0.05). Genes expressed at >1.0 Read(s) Per Kilobase of transcript per Million mapped reads (RPKM) in at least one GTEx tissue were included. Cell lines: KBM7- human myeloid leukaemia; K562- chronic myelogenous leukaemia; IMR90- human foetal lung; HUVEC-human umbilical vein endothelial cells; NHEK- primary normal human epidermal keratinocytes. (Supplementary File 1).

**Supplementary Table 3.** Summary of the functional prediction of T1D-associated SNPs

| SNP Name | Chromosome | Position | Reference allele | Alternate allele | DeepSEA score |
| --- | --- | --- | --- | --- | --- |
| rs2476601 | chr1 | 114377568 | A | G | 1.05E-03 |
| rs4625 | chr3 | 49572140 | A | G | 3.12E-03 |
| rs2836882 | chr21 | 40466570 | G | A | 5.28E-03 |
| rs5753037 | chr22 | 30581722 | C | T | 6.57E-03 |
| rs602662 | chr19 | 49206985 | G | A | 1.64E-02 |
| rs17696736 | chr12 | 112486818 | A | G | 1.68E-02 |
| rs1990760 | chr2 | 163124051 | C | T | 1.84E-02 |
| rs17388568 | chr4 | 123329362 | G | A | 1.87E-02 |
| rs12708716 | chr16 | 11179873 | A | G | 2.20E-02 |
| rs2153977 | chr1 | 114080071 | C | T | 2.27E-02 |
| rs2542151 | chr18 | 12779947 | G | T | 2.36E-02 |
| rs2903692 | chr16 | 11238783 | G | A | 2.49E-02 |
| rs1270942 | chr6 | 31918860 | A | G | 2.55E-02 |
| rs743777 | chr22 | 37551607 | A | G | 2.62E-02 |
| rs3184504 | chr12 | 111884608 | T | G | 2.68E-02 |
| rs10137082 | chr14 | 23840033 | C | T | 2.74E-02 |
| rs3024505 | chr1 | 206939904 | G | A | 2.84E-02 |
| rs3788013 | chr21 | 43841328 | C | A | 3.03E-02 |
| rs7804356 | chr7 | 26891665 | T | C | 3.97E-02 |
| rs10786436 | chr10 | 100300182 | C | T | 4.15E-02 |
| rs614226 | chr12 | 120974510 | C | T | 4.78E-02 |
| rs71597855 | chr4 | 54656437 | G | A | 4.80E-02 |
| rs10517086 | chr4 | 26085511 | G | A | 4.86E-02 |
| rs550448 | chr7 | 28229042 | G | A | 4.87E-02 |
| rs10186458 | chr2 | 20598510 | A | C | 4.93E-02 |

Summary table of the sequence-based deep learning-based sequence analyzer─DeepSEA (Zhou and Troyanskaya, 2015) functional prediction for the 113 significant SNP-eQTLs. The DeepSEA algorithm integrates a training set of genome-wide chromatin profiles based on the Roadmap Epigenomics and ENCODE datasets (Dunham et al., 2012; Kundaje et al., 2015). A functional significance score of <0.05 is considered significant for annotating potentially regulatory SNPs (Zhou and Troyanskaya, 2015). The full set of functional prediction scores (for all 180 SNPs associated with T1D) can be found in Supplementary File 1.


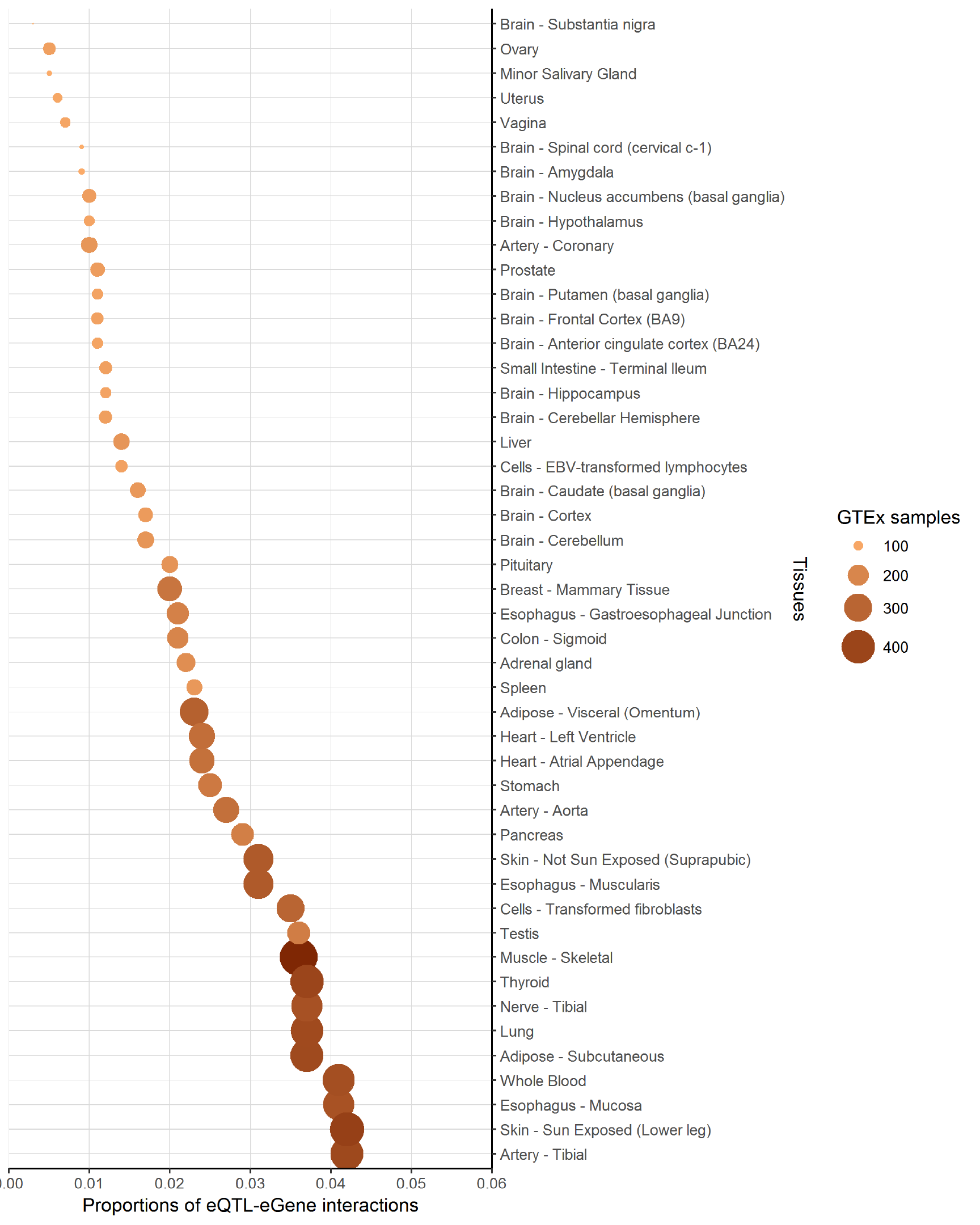


**Supplementary Figure 1. The distribution of disease-associated eGenes across human tissues.** The graph shows the proportions of eQTL-eGene interactions across tissues (x-axis) in comparison to number of GTEx tissue samples (circles). The enrichment of eGenes in tissues and cell types, such as whole blood, pancreas, thyroid, adipose, and skeletal muscle strongly suggests that T1D-associated variants in regulatory regions have tissue-specific effects since many regulatory regions have also been shown to act in a tissue-specific manner (*e.g*. (Ernst et al., 2011)). The relationship between genetic variation, gene expression, and T1D phenotype does not correspond directly to the number of tissue samples in the GTEx database. Genes expressed at >1.0 Read(s) Per Kilobase of transcript per Million mapped reads (RPKM) were included in this analysis (GTEx version 7, accessed on November 19, 2017). (Supplementary File 1).

**Supplementary references**

Dunham, I., Kundaje, A., Aldred, S. F., Collins, P. J., Davis, C. A., Doyle, F., et al. (2012). An integrated encyclopedia of DNA elements in the human genome. *Nature* 489, 57–74. doi:10.1038/nature11247.

Ernst, J., Kheradpour, P., Mikkelsen, T. S., Shoresh, N., Ward, L. D., Epstein, C. B., et al. (2011). Mapping and analysis of chromatin state dynamics in nine human cell types. *Nature* 473, 43–49. doi:10.1038/nature09906.

Kundaje, A., Meuleman, W., Ernst, J., Bilenky, M., Yen, A., Heravi-Moussavi, A., et al. (2015). Integrative analysis of 111 reference human epigenomes. *Nature* 518, 317–330. doi:10.1038/nature14248.

Zhou, J., and Troyanskaya, O. G. (2015). Predicting effects of noncoding variants with deep learning–based sequence model. *Nat. Methods* 12, 931–934. doi:10.1038/nmeth.3547.
